# Cyclical medication management interventions in health care settings: A systematic review

**DOI:** 10.1002/lrh2.70005

**Published:** 2025-02-26

**Authors:** Isabelle Meulenbroeks, Crisostomo Mercado, Rachel Urwin, Karla Seaman, Anna Kelly, Osman Qadri, Johanna Westbrook

**Affiliations:** ^1^ Australian Institute of Health Innovation Macquarie University North Ryde New South Wales Australia

**Keywords:** learning health systems, medication management, plan do study act, systematic review

## Abstract

**Introduction:**

It is estimated that one in 30 patients experiences at least one preventable medication‐related harm while receiving care. Cyclical medicine improvement interventions, where health systems continuously collect data, implement prescribing/dispensing interventions, review outcomes, and revise the intervention, have demonstrated health outcome improvements in a range of health care settings. This systematic review aimed to synthesize information on the characteristics and outcomes of cyclical medication management interventions.

**Methods:**

Five databases were systematically searched for cyclical medication management interventions from 2000 to 2023. Studies were screened in a two‐step process: title/abstract and full‐text screening. All intervention, population, and outcome data were extracted. Intervention data were thematically categorized, and outcome data were categorized using Proctor's framework. The quality of data was assessed using the Mixed Methods Appraisal Tool (MMAT).

**Results:**

Forty‐five cyclical interventions from 46 publications were included. Most interventions studied cyclical medication management interventions in hospital settings (80%, *n* = 37) and utilized the plan‐do‐study‐act framework to guide intervention design (64%, *n* = 29). Cyclical medication management interventions comprised multiple components (mean 2.4 components), with common components including practice standardization (*n* = 23), clinician feedback (*n* = 20), and clinician education (*n* = 18). One hundred and twenty‐two outcome measures were extracted and categorized as implementation (*n* = 77), service (*n* = 41), and patient outcomes (*n* = 4). The quality of many publications was poor; 8 publications could not be scored or scored 0 on the MMAT, and the remaining publications scored on average (mean) 60% on the MMAT.

**Conclusion:**

Cyclical medication management interventions show weak evidence that they can be implemented successfully and improve health system and service outcomes. Significant further research and health system structuring are required to address the quality issues surrounding cyclical medication management implementation and reporting.

## 1 INTRODUCTION

Internationally, health care systems aim to deliver high‐quality health care where the care delivered is safe, effective, patient‐centered, timely, efficient, and equitable.^1^ However, many health systems fall short of their aim. Globally, it is estimated that 30% of health care is low value, producing care that costs the health system but may not benefit the patient, and 10% of health care results in patient harm.^2^ Historically, efforts to bridge the gap between current practice and high‐quality health care have been unidirectional. For example, a group of researchers conducts a study on an intervention to improve health care quality, publishes the results, and waits for the results to be passively incorporated into practice to improve care quality in the health system. However, this unidirectional approach to improving the health care system is slow and does not yield health care improvement results specific to the local context.^3^, ^4^

Health systems that continuously collect and analyze their own data and implement or modify care accordingly may be better positioned to achieve high‐quality health care compared with those that rely on traditional research to practice translation processes.^5^ Afterall, rapid knowledge to practice loops and cyclical improvement frameworks have been used successfully for decades to improve health care quality and health system resource use. For example, in the 1980s, US health administrators used the plan‐do‐study‐act (PDSA) framework to understand medical coding errors and implemented interventions to improve accuracy in billing codes.^6^ In the 2000s, a third of US hospitals, in a survey of 56 organizations, were using the six‐sigma framework, another cyclical quality improvement framework, to collect data on current care processes and errors and implement interventions to improve care timelines and medical error rates.^7^ In the 2020s, care quality improved in an Australian university health system and in an international bowel disease network by developing health care staff knowledge and practice using a learning health system (LHS) framework.^8^ The LHS framework builds on earlier adaptations of cyclical improvement frameworks to harness the potential of routinely collected data in electronic health records and develop a culture of learning in the health system. While cyclical improvement frameworks implemented have demonstrated improved health care quality in past applications, they are not yet in routine practice in health care settings, and there are few high‐quality examples of them in peer‐reviewed literature.^9^ The lack of empirical evidence on effective cyclical improvement frameworks leaves the traditional, unidirectional method of research as the default method of health system care quality improvement.

Effective strategies are urgently required to improve medication management quality in health systems globally, as poor medication management is the largest contributor to preventable harm.^10^, ^11^ It is estimated that one in 30 patients experiences at least one preventable medication‐related harm while receiving care; more than a quarter of these incidents are considered severe or life‐threatening.^12^ Past research has indicated that continuous improvement frameworks, such as the PDSA and LHS, are effective at improving medication‐related health care quality, as they decrease the incidence of medication errors and adverse medication‐related harm.^13^, ^14^, ^15^ However, it is unclear whether cyclical improvement interventions consistently improve key measures of health care quality, such as patient health outcomes and health system resources. There are no syntheses available of cyclical improvement framework applications that aim to improve medication management quality. To inform the future application of cyclical medication management interventions, we conducted a systematic review that aimed to synthesize the characteristics of and outcomes resulting from peer‐reviewed applications of cyclical medication management interventions.

## 2 METHODS

### 2.1 Protocol

The planning and the reporting of this review followed the PRISMA guidelines.^16^ The review followed a protocol registered with PROSPERO (CRD42022333278).

### 2.2 Search strategy

The search strategy was developed by the research team in consultation with a clinical librarian. The databases searched included Cochrane Library, CINAHL Complete, Ovid MEDLINE, Scopus, and Ovid EMBASE. The search used synonyms for “leaning health system” and “medication management” and was restricted to only include journal article publications written in the English language from 2000 to August 2023. Publication date limits were applied to ensure that the review captures and can be translated to current practice. A full outline of search strategies can be found in Table A1. All collected publications were merged in the reference manager EndNote Version 20.^17^ Duplicates were removed before screening.

### 2.3 Inclusion and exclusion criteria

Articles were included if they were: (1) published in English, (2) peer‐reviewed journal articles, (3) published from 2000–present, (4) empirical research (gray literature and opinion/think pieces were excluded), and (5) investigated the effects of a cyclical, continuous improvement program on medication management within a health care setting. A health care setting was defined as any place or service where health care is provided (e.g., aged care, hospital, general practice). A cyclical, continuous improvement program was defined as any intervention that (a) was aimed at seeking improvement and positive change among program participants, (b) consisted of multiple improvement cycles (i.e., ≥2 cycles of coupling evidence generation with evidence application), and (c) utilized a multidisciplinary approach to learning (i.e., >1 professional specializations involved in approach). Publications with interventions that did not meet all aspects of these criteria (e.g., one‐off interventions with only one improvement cycle) were excluded. Due to the limited number of relevant publications, low publication quality was not a reason for article exclusion.

### 2.4 Selection and data collection processes

Publication screening followed two stages: title abstract and full‐text screening. Screening was conducted in Rayyan,^18^ an artificial intelligence‐supported mobile and web‐based application for systematic reviews, by three reviewers (KS, AK, RU). To ensure inter‐rater reliability, blinded review was conducted on a sample of 5% of publications between KS, AK, and RU at each screening stage, resulting in an average agreement rate of 96%. The remaining 95% of publications were divided among the research team to review. Weekly team meetings were held to discuss uncertainties in screening. During screening, relevant publications that did not meet the inclusion criteria (e.g., systematic reviews) were noted for snowballing purposes.

### 2.5 Data extraction

Two investigators (IM, CM) independently extracted the data to a purpose‐designed Microsoft Excel 2016 spreadsheet, which was then verified by one investigator (OQ). The following data items were extracted: aim, country, setting, study design, data source, control group, summary of research participants, summary of interventions, professional groups involved, evidence base for intervention, implementation framework, length of the interventions, sustainability plan, and outcomes. All outcomes within included papers were extracted.

### 2.6 Quality assessment

Critical appraisal of included publications was independently performed by two investigators (IM, CM) via the Mixed Methods Appraisal Tool (MMAT) version 2018.^19^ This tool was specifically developed for evaluating multiple intervention designs, including qualitative, quantitative, and mixed‐method studies, consisting of a checklist (Part I) and an explanation of the criteria (Part II). The response to each question is “yes” “no” or “can't tell”.^19^ A percentage of “yes” responses in every domain was used to summarize the MMAT scores. The two reviewers (IM, CM) applied the MMAT to include publications, and conflicts in the critical appraisal were discussed and resolved in regular team meetings.

### 2.7 Synthesis

**FIGURE 1:**
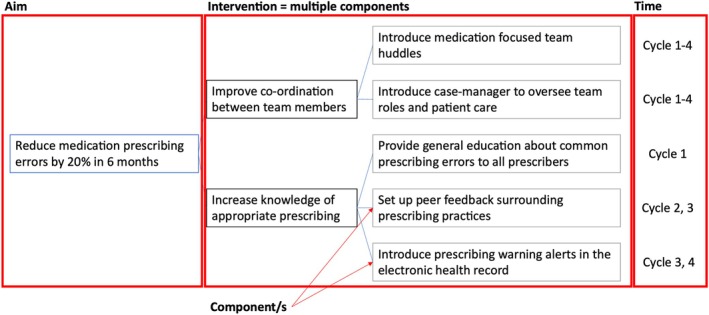
Visualization of intervention complexity.

Describing the intervention is complicated as cyclical interventions are commonly made up of many components that help to achieve the overall goal. An example of intervention complexity is presented in Figure 1. In this review, key intervention components were thematically grouped and synthesized to convey key methods used in medication management cyclical interventions.

Other features of the intervention such as country, setting, duration, and underpinning framework were descriptively analyzed (e.g., count, mean). Intervention outcomes were narratively synthesized using three categories, implementation, service, and patient outcomes (guided by Proctor's framework) to describe outcomes related to the introduction of the intervention, changes to the delivery of health care, and benefit or harm for the patient respectively (Table 1).^20^ Qualitative outcome results, and cases where both qualitative and quantitative results are summarized together, are labeled as “positive” or “negative” based on the study's main aim. For example, if a study aimed to explore the acceptability of a medication intervention among staff and interview results showed staff did accept the intervention, data extractors marked this as a “positive” qualitative outcome.

**TABLE 1:** Outcome categories and descriptions.

Outcome category	Definition
Implementation	Measures of intention such as the intention to implement a new intervention (early‐stage implementation), the degree to which the intervention was adhered to (mid‐stage implementation), or intention to sustain the intervention beyond the trial period (late‐stage implementation).
Service	Measure of observable effect on care delivery, for example, safety incidents, wait times, waste reduction, equitable care provision that does not vary due to personal characteristics, and care in line with best practice.
Patient	Measures of effect on people receiving care, for example, satisfaction and health outcomes

## 3 RESULTS

**FIGURE 2:**
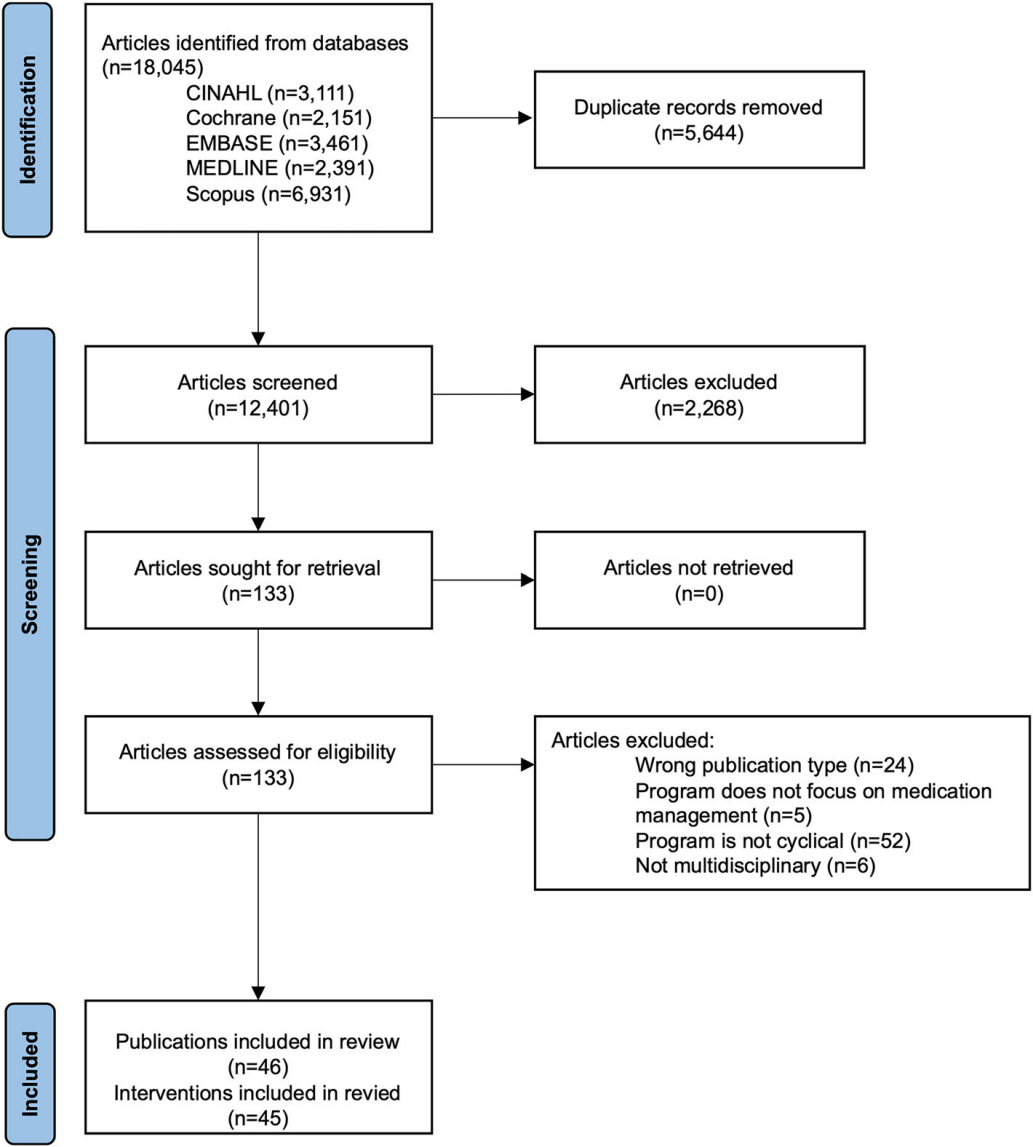
PRISMA flow diagram.

The search identified 18 045 entries from the selected databases (CINAHL: 3111, Cochrane: 2151, EMBASE: 3461, Medline: 2391 and Scopus: 6931). After the removal of duplicates, 12 401 publications were screened by the title and abstract. In total, 133 publications were retained for full‐text screening. Forty‐six publications, describing 45 interventions, met the inclusion criteria and were included in the review (Figure 2).

### 3.1 Quality appraisal results

Three publications were rated zero due to the lack of a description of the methods used.^21^, ^22^, ^23^ Five publications could not be appraised using MMAT, as initial MMAT criteria were not met (clear research question and study design to address research question).^24^, ^25^, ^26^, ^27^, ^28^ The remaining 37 publications received a score between 20% and 100% with a mean and median MMAT score of 60%. Eight publications achieved 100% using the MMAT.^29^, ^30^, ^31^, ^32^, ^33^, ^34^, ^35^, ^36^ The 29 publications^37^, ^38^, ^39^, ^40^, ^41^, ^42^, ^43^, ^44^, ^45^, ^46^, ^47^, ^48^, ^49^, ^50^, ^51^, ^52^, ^53^, ^54^, ^55^, ^56^, ^57^, ^58^, ^59^, ^60^, ^61^, ^62^, ^63^, ^64^, ^65^ with scores <100% frequently lost points because outcome data were incomplete (*n* = 13), confounders were not accounted for (*n* = 15), and the intervention was not administered as intended (*n* = 12). A complete breakdown of quality appraisal results is available in Table A2.

### 3.2 Setting

The majority of cyclical interventions were conducted in the United States (60%; *n* = 27),^23^, ^24^, ^25^, ^26^, ^27^, ^28^, ^29^, ^30^, ^31^, ^34^, ^35^, ^41^, ^42^, ^43^, ^46^, ^47^, ^49^, ^50^, ^51^, ^53^, ^54^, ^57^, ^58^, ^59^, ^61^, ^65^, ^66^ followed by the United Kingdom (16%; *n* = 7),^21^, ^22^, ^33^, ^40^, ^45^, ^56^, ^60^, ^62^, ^67^ Brazil (5%; *n* = 2),^37^, ^52^ and France (5%; *n* = 2).^32^, ^38^ In addition, one intervention was conducted in each of the following countries: Australia,^44^ Canada,^48^ Ireland,^40^ India,^63^ Saudi Arabia,^64^ New Zealand,^39^ and Uganda (Table 2).^57^

**TABLE 2:** Study characteristics.

Study	Setting	Participants	Intervention aim	Outcomes
Alghamdi 2023^64^	Defense hospitals, Saudi Arabia	Not reported	Improve unintentional medication discrepancy at admission by 50%	Number of patients with outstanding unintentional medication discrepancy at admission and discharge
Caixeta 2020^37^	Hospital, Brazil	712 patients	Improve compliance with cardiovascular quality indicators	Length of stay; door to balloon time; proportion of patients prescribed cardiovascular post‐operative medication at discharge
Cass 2013^24^	Local Health District outpatient clinics, United States	Not reported	Improve tuberculosis treatment indicators	Performance improvement plans completed
Curatolo 2015^38^	Hospital, France	246 patients	Improve medication reconciliation	Percentage of patients with complete medication history; unintended medication discrepancy
Dabrowski 2021^39^	Hospital, New Zealand	Not reported	Improve medication reconciliation	Percentage of medication reconciliation completed and partially completed
Devarajan 2022^65^	Childrens hospital, United States	399 hospital staff	Decrease prescription errors by 20%	Medication error rate; medication error rate requiring pharmacist intervention
Egan 2012^40^	Hospital, Ireland	75 patients	Revise hospital processes to make it easier for staff to carry out dosing and monitoring	Proportion of patients who received therapy in line with the new protocol
Fortney 2012^41^	Outpatient clinics, United States	3296 patients	Test the feasibility of using quality improvement principles to facilitate collaborative care management	Depression symptom severity; adoption rate; proportion of patients who had an encounter with a care manager; adherence to the intervention; follow‐up rate; cost; intention to sustain the intervention
Gavriloff 2012^25^	Hospital, United States	Not reported	Increase adherence to medication safety software	Adherence to medication safety software
Glenn 2019^42^	Childrens hospital, United States	312 patients	Implement a premedication protocol for intubation	Compliance with the protocol; time to intubation; intubation effectiveness; complications
Gordon 2012^21^	Childrens hospital, UK	26 hospital staff	Introduce prescribing feedback to enhance error awareness	Medication error rate; patient safety attitudes
Hanifin 2020^26^	Hospital, United States	Not reported	Integrate patient safety culture to reduce medication rate	Medication error rate; near‐miss medication error rate
Hatoun 2016^43^	Childrens hospital, United States	102 patients	Increase the proportion of patients who are discharged with medications in hand	Emergency department presentation; discharged with medications in hand
Hession‐Lab 2011^27^	Childrens health centre, United States	560 staff	Implement a reporting system to facilitate data collection and analysis of medication errors	Barriers to uptake; number of severe events
Hickey 2016^44^	Hospital, Australia	280 patients	Improve medication titration in heart failure patients by improving communication	Proportion of patients who had titration plan and who received target doses
Jeffries 2018^45^	Primary care facilities, UK	22 staff	Identify how a pharmacist‐led intervention for potentially hazardous prescribing could be embedded into everyday practice	Perceptions of ease of use
Jennings 2008^29^	Hospital system, United States	1222 patients	Reduce the incidence of anticoagulant medication‐related adverse events	Bleeding event rates; anticoagulant adverse drug event rates; rate of thrombotic events; bleeding and thrombotic reactions; reliability of hospital anticoagulation medication safety systems
Jones 2004^46^	Hospitals, United States	Not reported	Implement voluntary medication error reporting program	Proportion of errors reported
Joshi 2002^28^	Hospital system, United States	Not reported	Demonstrate benefits of error reporting intervention	Medication errors
Keogh 2016^47^	Hospital and speciality practices, United States	Not reported	Develop and implement a medication reconciliation process	Medication reconciliation performance
Kern 2017^30^	Ambulatory practice, United States	Not reported	Assess performance in medication reconciliation	Proportion of: Medication reconciliation completed; missing dose or frequency of medication; duplicate medication; patient handouts given; compliance with intervention; printed medication list available at visit
Kuhlmann 2013^31^	Hospital, United States	24 patients	Identify pitfalls in compliance with childhood asthma care measures and increase compliance to >90%	Compliance with childhood asthma care measures
Leach 2016^22^	Hospital, UK	Not reported	Reduce prescribing error rate by developing and implementing new standards	Medication error rate; attitudes towards intervention
Lees 2011^48^	Hospitals, Canada	Not reported	Implement medication reconciliation and evidence‐based care for myocardial infarction	Adverse drug events; medication reconciliation rate; acute myocardial infarction rate
Lesar 2003^49^	Hospitals, United States	Not reported	Implement and evaluate medication use system safety improvements	Medication safety self‐assessment scores
Ligi 2010^32^	Neonatal centre, France	Not reported	Assess the impact of continuous reporting and prevention strategies for severe iatrogenic events	Proportion of patients with drug infusion rate errors; severe cutaneous injuries; unplanned exudations; invasive procedures; catheter related infections; severe iatrogenic events
McFadzean 2023^62^	General practice, UK	Not reported	Improve direct oral anticoagulant monitoring	Number of medication reviews completed
Meisel 2007^50^	Hospital, United States	Not reported	Reduce the rate of serious narcotic oversedation	Number of serious adverse drug events
Mondal 2022^63^	Hospital, India	124 patients	Assess medication errors and using a quality improvement program reduce errors	Number of prescribing errors
Mutter 2003^23^	Hospital, United States	Not reported	Reduce medication errors	Number of medication errors
Patel 2020^51^	Childrens hospital, United States	Not reported	Analyses prescribed seizure medication doses to identify inappropriate low dose prescriptions	Compliance; provider prescribed and signed low‐dose rescue medication
Pereira 2020^52^	Hospital, Brazil	Not reported	Implement quality improvement program to reduce oral medication preparation and administration errors	Proportion of oral medications correctly prepared (crushed, titrated, mixed) and administered (feeding tube obstruction)
Ramsay 2014^33^	Hospitals, UK	634 patients, 49 hospital staff	Evaluate the effect of ward‐level medication safety score card	Staff acceptability; percentage of patients with allergy documentation, drugs omitted from record, and appropriately colored wrist band; percentage of patients with inappropriate own drugs in patient locker
Rappaport 2011^34^	Outpatient children's hospital, United States	Not reported	Implement electronic medical record quality improvement project to improve medication reconciliation	Documentation of medication reconciliation
Robbins 2013^53^	Community health centre, United States	Not reported	Integrate pharmacy services to enhance patient safety	Medication reconciliation rate; obesity screening and follow‐up documentation; adverse drug event reporting; clinical pharmacy service use
Rungvivatjarus 2020^35^	Childrens hospital, United States	Not reported	Increase medication reconciliation at hospital admission	Percentage of admission encounters with complete medication reconciliation overall and across 13 drug classes
Russ 2020^54^	Childrens intensive care unit, United States	Not reported	Reduce errors in admission medication reconciliation by 50%	Medication reconciliation error rate
Schnipper 2018^66^	Hospitals, United States	1648 patients	Reduce potentially harmful medication discrepancies	Medication discrepancies with potential for harm and all discrepancies
Styles 2019^56^, ^60^	Childrens ward, UK	Not reported	Increase staff awareness and culture around medication errors and reduce prescribing errors by 20%	Prescribing error rate
Subramanyam 2016^57^	Childrens hospital, United States	Not reported	Implement two‐person verification system before medication administration	Proportion of medication double‐checked; medication error
Sullivan 2013^58^	Neonatal intensive care unit, United States	Not reported	Report the development of a prescribing error feedback	Number of days between narcotic and antibiotic prescribing errors; overall rate of prescribing errors; rate of antibiotic prescribing errors
Trap 2018^36^	1499 facilities, Uganda	Not reported	Assess the impact of introducing medicines management supervisors whose role it is to oversee medication stock management, storage management, ordering, dispensing, prescribing, and dispensing quality	supervision, performance assessment, and recognition strategy scores
Triller 2014^59^	Long term care facilities, United States	669 patients	Assess the impact of warfarin safety intervention on objective quality measures	Proportion of residents receiving INR testing; time in therapeutic range; mean INR; proportion of INRs between 2.3 and 2.7
Trivedi 2020^67^	Hospital, UK	Not reported	Increase the rate of medication reconciliation	Medication reconciliation rate
Wong 2018^61^	Outpatient clinic, United States	58 patients	Improve adherence to quality metrics	Opioid agreement; toxicology screening; number of providers; annual office visits per patient; opioid risk score

Abbreviation: INR, international normalized ratio.

### 3.3 Population

Most interventions were conducted in hospital populations (80%, *n* = 37).^21^, ^22^, ^23^, ^25^, ^26^, ^27^, ^28^, ^29^, ^31^, ^32^, ^33^, ^34^, ^35^, ^36^, ^37^, ^38^, ^39^, ^40^, ^42^, ^43^, ^44^, ^46^, ^47^, ^48^, ^49^, ^50^, ^51^, ^52^, ^54^, ^55^, ^56^, ^57^, ^58^, ^60^, ^63^, ^64^, ^65^, ^67^ Nineteen interventions explored continuous improvement cycles in populations with specific characteristics, such as pediatric populations (*n* = 17),^21^, ^22^, ^27^, ^31^, ^32^, ^34^, ^35^, ^42^, ^43^, ^51^, ^54^, ^56^, ^57^, ^58^, ^60^, ^63^, ^65^, ^67^ veterans (*n* = 1),^64^ and women (*n* = 1).^47^ The remaining interventions were conducted in adult populations.

### 3.4 Intervention

#### 3.4.1 Theoretical design

Intervention design was frequently underpinned by the PDSA framework and was the most utilized cyclical framework to guide intervention design (64%, *n* = 29).^22^, ^25^, ^27^, ^29^, ^31^, ^34^, ^35^, ^37^, ^38^, ^39^, ^41^, ^42^, ^43^, ^44^, ^47^, ^51^, ^52^, ^53^, ^54^, ^56^, ^57^, ^58^, ^60^, ^61^, ^62^, ^63^, ^64^, ^65^, ^67^ In three interventions, the PDSA framework was supplemented by additional frameworks such as Drug Usage Evaluation (DUE) (*n* = 1),^29^ Failure Modes and Effects Analysis (FMEA) (*n* = 2),^29^, ^48^ Root Cause Analysis (RCA) (*n* = 1),^29^ and Model for Improvement (*n* = 1).^27^ FMEA was also applied alone in one intervention.^23^ Other frameworks applied to intervention design included Six Sigma (*n* = 1),^40^ LHS (*n* = 1),^46^ quality improvement (*n* = 1),^28^ and Medications At Transition and Clinical Handoffs (MATCH) (*n* = 1).^30^ Ten interventions did not specify or use a framework to support the design of the intervention; however, they still described intervention cycles.^21^, ^24^, ^26^, ^32^, ^33^, ^36^, ^49^, ^50^, ^55^, ^59^

#### 3.4.2 Aims

Thirty interventions broadly aimed to improve safe prescription, administration, and/or monitoring of medications during an episode of care,^21^, ^22^, ^23^, ^24^, ^25^, ^26^, ^27^, ^29^, ^31^, ^32^, ^33^, ^36^, ^37^, ^40^, ^41^, ^42^, ^44^, ^45^, ^46^, ^49^, ^50^, ^51^, ^52^, ^56^, ^57^, ^59^, ^61^, ^62^, ^65^ while 14 focused on medication reconciliation,^30^, ^34^, ^35^, ^38^, ^39^, ^47^, ^48^, ^53^, ^54^, ^58^, ^63^, ^64^, ^66^, ^67^ and two focused on the transition or discharge of medications.^43^, ^44^ The aim of each intervention is summarized in Table 2.

#### 3.4.3 Components

In this review, interventions (*n* = 45) comprised an average (mean) of 2.4 and a median of 2 different components (Figure 1) that were grouped into eight themes (Table 3). Thematic intervention components identified in this review included the implementation of standardized practices (*n* = 23),^23^, ^25^, ^28^, ^30^, ^31^, ^34^, ^35^, ^40^, ^42^, ^43^, ^47^, ^48^, ^49^, ^52^, ^53^, ^54^, ^57^, ^59^, ^61^, ^63^, ^65^, ^66^, ^67^ clinician feedback (*n* = 20),^21^, ^22^, ^24^, ^27^, ^28^, ^33^, ^34^, ^35^, ^37^, ^39^, ^41^, ^45^, ^46^, ^51^, ^53^, ^56^, ^58^, ^59^, ^64^, ^65^ educating clinicians (*n* = 18),^23^, ^25^, ^27^, ^30^, ^31^, ^32^, ^35^, ^43^, ^47^, ^49^, ^52^, ^54^, ^57^, ^59^, ^63^, ^65^, ^66^, ^67^ adjustment of governance structures (*n* = 13),^24^, ^26^, ^29^, ^32^, ^36^, ^37^, ^41^, ^44^, ^47^, ^49^, ^51^, ^53^, ^64^ defining existing roles in the multidisciplinary team (*n* = 11),^30^, ^35^, ^37^, ^40^, ^42^, ^43^, ^50^, ^53^, ^61^, ^63^, ^64^ creation of new roles (*n* = 10),^22^, ^25^, ^27^, ^29^, ^36^, ^48^, ^51^, ^53^, ^57^, ^62^ and strengthening communication between team members (*n* = 10).^25^, ^37^, ^44^, ^47^, ^50^, ^53^, ^56^, ^59^, ^61^, ^64^ Overall, eight interventions included patients and/or their caregivers either through consultation in intervention design or inclusion as participants, such as receiving education.^32^, ^34^, ^44^, ^46^, ^49^, ^55^, ^62^, ^67^ Four of these included patients and caregivers through an education component (*n* = 4).^32^, ^49^, ^55^, ^62^

**TABLE 3:** Thematic components of cyclical medication safety continuous improvement interventions.

Theme	Description
Governance structures	Managerial team or staff included to monitor and intervene in medication management intervention. For example, organizational meetings to review medication safety and patient outcome measures.
Clinician feedback	Clinicians provided feedback on medication safety. For example, alerts of prescribing error in real time for the clinician, individualized reports for the clinician regarding the type and number of errors with ways to improve, and peer‐review feedback sessions with other clinicians such as a pharmacist.
Standardized practices	Existing standardized process/policies/pathways/structures improved or new ones developed. For example, implementation of a new electronic medical record system for medication prescribing and administration, and re‐structuring of medication lists on intravenous pumps to reduce time to select the correct medication.
New job creation	The creation of a new job or hire of additional staff to manage/implement the intervention. For example, a case manager to oversee medication management.
Defining existing roles	Outlining roles of team members in medication management (without hiring or creating new role). For example, describing who in a resuscitation team is responsible for what.
Educate patients	Education (materials, cues, verbal communication etc.) provided to patients. For example, tools to support caregivers to participate in medication reconciliation.
Educate clinicians	Clinicians prescribing, administering, or monitoring the medication are broadly educated through non‐personalized education services such as in services, posters, and dissemination of industry guidelines.
Strengthen communication	Efforts to strengthen information transfer between clinicians involved in patient care. For example, implementation of team huddles.

#### 3.4.4 Team members

Due to the inclusion criteria, all interventions included in this review were implemented by a multidisciplinary team. Among the 45 interventions, doctors were the most frequently included professional discipline in multidisciplinary teams (*n* = 40),^21^, ^22^, ^23^, ^24^, ^28^, ^29^, ^30^, ^31^, ^32^, ^33^, ^34^, ^35^, ^37^, ^38^, ^39^, ^40^, ^41^, ^42^, ^43^, ^44^, ^45^, ^46^, ^47^, ^48^, ^49^, ^50^, ^51^, ^53^, ^54^, ^55^, ^56^, ^57^, ^58^, ^59^, ^61^, ^62^, ^63^, ^64^, ^65^, ^67^ followed by nurses (*n* = 38)^22^, ^23^, ^24^, ^25^, ^26^, ^27^, ^28^, ^30^, ^31^, ^32^, ^33^, ^34^, ^35^, ^37^, ^38^, ^40^, ^41^, ^42^, ^43^, ^44^, ^45^, ^46^, ^47^, ^48^, ^49^, ^50^, ^51^, ^52^, ^54^, ^56^, ^57^, ^58^, ^59^, ^61^, ^62^, ^63^, ^64^, ^67^ and pharmacists (*n* = 27).^22^, ^23^, ^25^, ^26^, ^29^, ^31^, ^33^, ^34^, ^35^, ^38^, ^39^, ^40^, ^42^, ^43^, ^44^, ^46^, ^49^, ^50^, ^53^, ^54^, ^55^, ^56^, ^58^, ^59^, ^64^, ^65^, ^67^ Twenty‐six interventions^24^, ^26^, ^29^, ^30^, ^31^, ^32^, ^34^, ^35^, ^36^, ^37^, ^40^, ^42^, ^46^, ^47^, ^48^, ^49^, ^50^, ^51^, ^52^, ^53^, ^54^, ^57^, ^58^, ^62^, ^64^, ^65^ included other professionals such as quality improvement consultants,^65^ epidemiologists,^32^ respiratory therapists,^42^ and laboratory personnel.^29^ Eighteen interventions also included administrative or management personnel in the multidisciplinary team.^22^, ^23^, ^24^, ^26^, ^27^, ^28^, ^29^, ^31^, ^32^, ^34^, ^46^, ^47^, ^49^, ^50^, ^51^, ^52^, ^53^, ^57^

#### 3.4.5 Intervention length

The total duration of the intervention was reported in 35 interventions (85.4%) with a range of 1–144 months, an average duration of 26.5 months, and a median of 18 months.^21^, ^24^, ^25^, ^26^, ^27^, ^29^, ^30^, ^31^, ^32^, ^33^, ^34^, ^35^, ^36^, ^37^, ^38^, ^40^, ^41^, ^42^, ^43^, ^44^, ^47^, ^48^, ^50^, ^51^, ^53^, ^54^, ^55^, ^56^, ^57^, ^58^, ^59^, ^60^, ^61^, ^62^, ^63^, ^64^, ^65^, ^67^ Twenty‐two interventions did not clearly state the number of improvement cycles that were conducted during the intervention timeline.^24^, ^25^, ^26^, ^27^, ^28^, ^29^, ^30^, ^34^, ^36^, ^37^, ^41^, ^42^, ^44^, ^45^, ^46^, ^47^, ^48^, ^49^, ^50^, ^55^ In the 23 studies that specified the number of improvement cycles, the number of cycles ranged from 2 to 54, with a mean of eight cycles and a median of three cycles.^21^, ^22^, ^31^, ^32^, ^33^, ^35^, ^38^, ^39^, ^40^, ^43^, ^51^, ^52^, ^53^, ^54^, ^56^, ^57^, ^58^, ^59^, ^60^, ^61^, ^62^, ^63^, ^64^, ^65^, ^67^

### 3.5 Outcomes

One hundred and twenty‐two outcome measures were reported among the 45 interventions. Of these outcomes, 77 were categorized as implementation outcomes,^21^, ^22^, ^24^, ^25^, ^27^, ^28^, ^30^, ^31^, ^33^, ^34^, ^35^, ^36^, ^37^, ^38^, ^39^, ^40^, ^41^, ^42^, ^43^, ^44^, ^45^, ^47^, ^48^, ^49^, ^51^, ^52^, ^53^, ^54^, ^55^, ^57^, ^59^, ^61^, ^62^, ^64^, ^67^ 41 as service outcomes,^21^, ^22^, ^23^, ^26^, ^27^, ^29^, ^32^, ^37^, ^38^, ^42^, ^43^, ^46^, ^48^, ^50^, ^53^, ^55^, ^56^, ^57^, ^58^, ^59^, ^60^, ^61^, ^63^, ^65^ and four as patient outcomes.^29^, ^37^, ^41^ No intervention enrolled a control group. Instead, where comparisons were made in outcome measures, baseline measures were used.

#### 3.5.1 Implementation

Among the 77 outcomes categorized as implementation outcomes, the majority (*n* = 67)^24^, ^25^, ^28^, ^30^, ^31^, ^33^, ^34^, ^35^, ^36^, ^37^, ^38^, ^39^, ^40^, ^41^, ^42^, ^43^, ^44^, ^47^, ^48^, ^49^, ^51^, ^52^, ^53^, ^54^, ^55^, ^57^, ^59^, ^61^, ^62^, ^64^, ^67^ focused on adherence to the intervention, an early‐mid stage implementation outcome. For example, the proportion of medicines prepared in accordance with a new quality improvement programme standard at certain time periods^52^ or the proportion of intubations that were compliant with a newly implemented medication sheet.^42^ Generally, studies reported good adherence to the intervention. Only three studies reported low adherence to components of the intervention.^39^, ^52^, ^67^ For example, patient education sheets were not discharged with the patient as intended in one medication reconciliation intervention.^30^

Another seven implementation outcomes focused on other early–mid‐stage implementation outcomes not related to adherence to the intervention.^21^, ^22^, ^27^, ^33^, ^41^, ^45^ For example, outcome measures of attitude, such as staff satisfaction with aspects of the intervention and perceptions of ease of use^33^ and barriers to uptake of the intervention.^27^ All seven remaining early–mid‐stage implementation outcomes were reported positively, in qualitative outcomes (*n* = 4),^22^, ^27^, ^33^, ^45^ or improved from baseline measures in quantitative outcomes (*n* = 3).^21^, ^41^

Three outcomes, from two interventions,^38^, ^41^ were outcomes that are typically measured later in the implementation process: cost (*n* = 1) and sustainability (*n* = 2) of the intervention. Across the two interventions, taking the best possible medication histories^38^ and multidisciplinary depression management,^41^ participants either sustained the intervention beyond the study period^38^ or expressed intent to continue the intervention after funding had ceased.^41^ Authors reported late‐stage outcomes positively, that is, they judged that the cost of the intervention was low and intent to sustain the intervention was high.

#### 3.5.2 Service outcomes

Forty‐one outcomes were categorized as service‐level outcomes. Many of the service outcomes focused on medication safety (*n* = 27).^21^, ^22^, ^23^, ^26^, ^27^, ^29^, ^32^, ^38^, ^42^, ^46^, ^48^, ^50^, ^53^, ^55^, ^56^, ^57^, ^58^, ^60^, ^63^, ^65^ For example, medication error rates at baseline and post‐intervention and the number of near misses.^21^, ^22^, ^23^, ^26^, ^58^ All medication safety outcomes reportedly improved with the intervention, except for one intervention which found no change in prescribing errors.^58^

All other service outcomes (*n* = 14)^29^, ^32^, ^37^, ^42^, ^43^, ^59^, ^61^ collected were measures of timeliness, such as door‐toto‐balloon time in myocardial infarction patients,^37^ and resource use, such as the number of emergency department presentations^43^ or invasive procedures.^32^ The 14 service outcomes not related to medication safety had mixed results. Five service outcomes did not change with the intervention; these included no change in length of stay^37^ and no change in the rate of intubations achieved to attempts.^42^ One outcome was poorer: the incidence rate of unplanned extubations increased significantly.^32^ Eight outcomes improved, such as 30‐day emergency department admissions following an intervention where patients were discharged with medications in hand^43^ and improved clinic utilization by patients attending scheduled follow‐up clinics following a workflow redesign for opioid management.^61^

#### 3.5.3 Patient outcomes

Four patient outcomes were extracted from three interventions.^29^, ^37^, ^41^ All were health‐related, as opposed to patient experience or satisfaction related, and all improved with the intervention. For example, compared with baseline measures, depressive symptoms improved in a veteran‐specific depression intervention,^41^ bleeding rates and the rate of thrombotic events improved in an intervention that aimed to reduce anticoagulant medication errors,^29^ and a study targeting key myocardial infarction care quality metrics reported an improvement in adjusted mortality.^37^

## 4 DISCUSSION

In this review, we found that cyclical medication management interventions are concentrated within the United States and in hospital settings. The cyclical interventions often utilized components such as educating clinicians, providing feedback to clinicians, and establishing protocols to improve medication management. Outcomes reported among these studies overwhelmingly focused on implementation outcomes, such as compliance with the intervention or satisfaction with the new process. Fewer studies reported evaluation of service outcomes or patient outcomes.

We identified that cyclical medication management interventions can be implemented successfully.^39^, ^52^, ^67^ However, we did not identify strong evidence to determine whether cyclical medication management interventions improve patient or service outcomes, as few studies reported or measured these. The strongest evidence identified was for service outcomes, specifically medication safety, as 26 of 27 medication safety outcomes reported an improvement in outcomes, such as prescribing and administrative errors.^21^, ^22^, ^23^, ^26^, ^27^, ^29^, ^32^, ^38^, ^42^, ^46^, ^48^, ^50^, ^53^, ^55^, ^56^, ^57^, ^58^, ^60^, ^63^, ^65^ There was weak but consistent evidence, due to the low quantity and quality of research, that cyclical medication management improved patient outcomes.^29^, ^37^, ^41^ Adverse events, or worse performance in patient health or service outcomes, were rarely reported in cyclical medication management interventions, suggesting that these interventions are often low risk. The improvements in patient safety and health outcomes identified in this review, with limited risk, suggest that cyclical medication interventions can improve care quality in health systems. However, the feasibility and clinical benefit of cyclical medication interventions require confirmation in further high‐quality research with comparator groups.

Characteristics of successful interventions that showed improvements in service and patient outcome measures could inform future research. Future cyclical medication management interventions should specifically outline a target population and sample size, duration, and number of improvement cycles included, and use a higher number of intervention components since successful interventions had a more robust study design and on average had (mean 2.8 vs. 2.4) more intervention components.^29^, ^32^, ^37^, ^42^, ^43^, ^61^ Common intervention components in successful interventions included: defining existing roles, governance structures, and standardized practices.^29^, ^32^, ^37^, ^42^, ^43^, ^61^ Successful interventions also focused on prescribing/administration of medications rather than discharge or transfer of medication‐related information.^29^, ^32^, ^37^, ^42^, ^43^, ^61^ However, the specific components and targets of the intervention need to be adapted to target research study aims.

The poor quality of studies identified in this review made it difficult to extract the findings and to show demonstrated benefits. Poor study quality may reflect difficulties authors experienced implementing the cyclical interventions. For example, many interventions in this review may not have been able to clearly define their included population as the population enrolled in the intervention evolved over time. For example, hypothetically, in Cycle 1 authors could have included all older adults in the intervention; however, after data collection and analysis, authors may update the inclusion criteria in Cycle 2 to all older adults with a heart condition to optimize resource use to the most at‐risk population. Poor study quality may also be the result of traditional research structures. For example, cyclical interventions may not receive the same funding as traditional rigid research methods, such as randomized control trials (RCT), limiting the resources that researchers must have to conduct the study, write, and publish the results (e.g., limiting researcher to adopt pre‐post design). Typical journal structures also do not accommodate cyclical interventions as they do not conform to the standard reporting guidelines. This may lead to the publication of many cyclical intervention results as perspective articles or case studies, as seen in our review, and limit the detail and type of information published. Ultimately, there is no gold standard guidance on how to design and disseminate information on cyclical improvement interventions that align with an LHS approach as there is for other study designs (e.g., the consolidated standards of reporting trials [CONSORT] for RCTs).^68^ In the future, cyclical intervention methods and reporting should be standardized to improve the design and dissemination of adaptive interventions.

## 5 LIMITATIONS

The findings of this review should be considered in the context of the following limitations. First, studies of poor quality were included in the review as there is limited evidence on cyclical medication management interventions. In addition, all studies were pre–post studies, which are poor indicators of effectiveness when compared with studies that enroll a comparator group. The inclusion of low‐quality evidence may have biased the results, likely towards reporting positive results due to underlying author bias in included interventions. Second, our search strategy may not have captured all possible cyclical medication management interventions, as not all studies describe their interventions using common frameworks or describe the cyclical nature of the intervention in the title or abstract of the publication, resulting in papers being missed in the search strategy or discarded in title abstract screening.^69^ Third, our review relied on subjective assessments and categorizations to synthesize the diverse data. The outcomes extracted were categorized using Proctor's framework, while intervention components were categorized using themes developed by the research team. In previous reviews, we have observed significant inconsistencies between researchers' categorizations when using Proctor's framework.^70^ Outcomes were further classified as having a “positive” or “negative” impact based on subjective assessments by researchers, as statistical evaluation was not consistently available (e.g., for qualitative data). These subjective assessments may have introduced inconsistencies and carried over biases from the original studies into the presentation of results. To minimize this risk, all categorizations and outcome assessments were reviewed by a single author.

## 6 CONCLUSIONS

Cyclical medication management interventions show weak evidence that they can be implemented successfully and improve health system and service outcomes. Based on successful interventions, future cyclical medication management interventions should use multiple (e.g., ≥3) intervention components and adopt a comprehensive study design. Significant further research and health system structuring are required to address the quality issues surrounding cyclical medication management implementation and reporting.

## FUNDING INFORMATION

This project was funded by an Australian NHMRC Partnership Project Grant with Anglicare, BaptistCare, Scalabrini, and ACSQHC (APP2006957). JIW is supported by an NHMRC Elizabeth Blackburn Leadership Fellowship (1174021). The NHMRC did not have any influence on the design of the study and the collection, analysis, and interpretation of data.

## CONFLICT OF INTEREST STATEMENT

The authors declare no conflicts of interest.

## Data Availability

All relevant data are contained within the manuscript and its appendices.

## Appendix

**TABLE A1:** Search strategy (date of search: 03/08/2023).

Database	Search field	Keywords/MeSH terms	Limits
CINAHL complete	Subject terms Keywords in title or abstract	**Cyclical continuous improvement programs:** (MH “Learning Health System”) (MH “Evaluation and Quality Improvement Program”) (MH “Benchmarking”) (MH “Process Assessment (Health Care)+”) (MH “Quality Assurance+”) TI (“Learning Health System*” or “learning system*” or roundtabl* or “knowledge to practice” or k2p or benchmark* or “quality indicator*” or “Continuous improvement*” or “Continuous quality improvement” or “Quality improvement” or “learning communit*” or “medication advisory*” or “outcome assessment*” or “process assessment*” or “quality assurance*” or “total quality management”) OR AB (“Learning Health System*” or “learning system*” or roundtabl* or “knowledge to practice” or k2p or benchmark* or “quality indicator*” or “Continuous improvement*” or “Continuous quality improvement” or “Quality improvement” or “learning communit*” or “medication advisory*” or “outcome assessment*” or “process assessment*” or “quality assurance*” or “total quality management”) **Medication management:** (MH “Medication Reconciliation”) (MH “Medication Compliance”) (MH “Medication Systems”) (MH “Medication Errors+”) (MH “Medication Management”) TI (“Medication management” or “Prescription drug management” or “Medication monitoring” or “Medication reconciliation” or “Medicine management” or “Electronic Medication Management” or EMM or eMeds or “Medication therapy management” or “Polypharmacy management*” or “Medication review” or “Medication Adherence” or “Medication Error*” or “Medication System*”) OR AB (“Medication management” or “Prescription drug management” or “Medication monitoring” or “Medication reconciliation” or “Medicine management” or “Electronic Medication Management” or EMM or eMeds or “Medication therapy management” or “Polypharmacy management*” or “Medication review” or “Medication Adherence” or “Medication Error*” or “Medication System*”)	Published date: 20000101 English language Publication Type: Journal Article
MEDLINE complete	Subject headings Keywords in title or abstract	**Cyclical continuous improvement programs:** (Learning health system* or learning system* or roundtable* or knowledge to practice or k2p or benchmark* or quality indicator* or Continuous improvement* or Continuous quality improvement or Quality improvement or learning communit* or medication advisory* or outcome assessment* or process assessment* or quality assurance* or total quality management).ti,ab. Learning Health System/ “outcome and process assessment, health care”/ or quality assurance, health care/ or benchmarking/ or Quality Improvement/ or Total Quality Management/ **Medication management:** (Medication management or Prescription drug management or Medication monitoring or Medication reconciliation or Medicine management or Electronic Medication Management or EMM or eMeds or Medication therapy management or Polypharmacy management* or Medication review or Medication Adherence or Medication Error* or Medication System*).ti,ab. Medication Therapy Management/ or Medication Errors/ or Medication Systems/ or Medication Adherence/ or “Medication Review”/ or Medication Reconciliation/	Language: English Date: 2000 Publication type: Journal article
Scopus	Title, abstract, keywords	**Cyclical continuous improvement programs:** TITLE‐ABS‐KEY (*“Learning health system*”* OR *“learning system*”* OR *roundtable** OR *“knowledge to practice”* OR *k2p* OR *benchmark** OR *“quality indicator*”* OR *“Continuous improvement*”* OR *“Continuous quality improvement”* OR *“Quality improvement”* OR *“learning communit*”* OR *“medication advisory*”* OR *“Quality Assuranc*”* OR *“Outcome Assessment”* OR *“Process Assessment”* OR *“Total quality management”*) **Medication management:** TITLE‐ABS‐KEY (*“Medication management”* OR *“Prescription drug management”* OR *“Medication monitoring”* OR *“Medication reconciliation”* OR *“Medicine management”* OR *“Electronic Medication Management”* OR *emm* OR *emeds* OR *“Medication therapy management”* OR *“Polypharmacy management*”* OR *“Medication Error*”* OR *“Medication System*”* OR *“Medication Adherence”* OR *“Medication Review*”*)	Language: English Date: 2000 Document type: article
Embase	Subject headings Keywords in title or abstract	**Cyclical continuous improvement programs:** (Learning health system* or learning system* or roundtable* or knowledge to practice or k2p or benchmark* or quality indicator* or Continuous improvement* or Continuous quality improvement or Quality improvement or learning communit* or medication advisory* or outcome assessment* or process assessment* or quality assurance* or total quality management).ti,ab. Learning Health System/ health care quality/ or benchmarking/ or clinical indicator/ or health care surveillance/ or medical error/ or performance measurement system/ or program evaluation/ **Medication management:** (Medication management or Prescription drug management or Medication monitoring or Medication reconciliation or Medicine management or Electronic Medication Management or EMM or eMeds or Medication therapy management or Polypharmacy management* or Medication review or Medication Adherence or Medication Error* or Medication System*).ti,ab. medication therapy management/ or medication compliance/ or medication error/	Language: English Date: 2000 Publication type: Article
Cochrane Library	Title, abstract, keywords MeSH descriptors	**Cyclical continuous improvement programs:** (“Learning health system” OR “learning system” OR roundtable OR “knowledge to practice” OR k2p or benchmark OR “quality indicator” OR “Continuous improvement” OR “Continuous quality improvement” OR “Quality improvement” OR “learning community” OR “medication advisory committee” OR “outcome assessment” OR “process assessment” OR “quality assurance” OR “total quality management”):ti,ab,kw MeSH descriptor: [Learning Health System] explode all trees MeSH descriptor: [Process Assessment, Health Care] explode all trees MeSH descriptor: [Quality Improvement] explode all trees MeSH descriptor: [Total Quality Management] explode all trees MeSH descriptor: [Benchmarking] explode all trees **Medication management:** (“Medication management” OR “Prescription drug management” OR “Medication monitoring” OR “Medication reconciliation” OR “Medicine management” OR “Electronic Medication Management” OR EMM or eMeds OR “Medication therapy management” OR “Polypharmacy management” OR “Medication review” or “Medication Adherence” or “Medication Error” or “Medication System”):ti,ab,kw MeSH descriptor: [Medication Therapy Management] explode all trees MeSH descriptor: [Medication Errors] explode all trees MeSH descriptor: [Medication Adherence] explode all trees MeSH descriptor: [Medication Reconciliation] explode all trees MeSH descriptor: [Medication Review] explode all trees MeSH descriptor: [Medication Systems] explode all trees	Date: 2000 Type: Cochrane trials

**TABLE A2:** Quality appraisal results.

	1.1	1.2	1.3	1.4	1.5	3.1	3.2	3.3	3.4	3.5	4.1	4.2	4.3	4.4	4.5	5.1	5.2	5.3	5.4	5.5	Percentage
Alghamdi 2023^63^						•	•	•	•	•											40
Caixeta 2020^36^						•	•	•	•	•											60
Curatolo 2015^37^											•	•	•	•	•						40
Dabrowski 2021^38^											•	•	•	•	•						80
Devarajan 2022^64^						•	•	•	•	•											80
Egan 2012^39^											•	•	•	•	•						60
Fortney 2012^40^						•	•	•	•	•											40
Glenn 2019^41^											•	•	•	•	•						20
Gordon 2012^20^											•	•	•	•	•						0
Hatoun 2016^42^											•	•	•	•	•						20
Hickey 2016^43^						•	•	•	•	•											80
Jeffries 2018^44^	•	•	•	•	•																80
Jennings 2008^28^						•	•	•	•	•											100
Jones 2004^45^											•	•	•	•	•						80
Keogh 2016^46^						•	•	•	•	•											60
Kern 2017^29^						•	•	•	•	•											100
Kuhlmann 2013^30^											•	•	•	•	•						100
Leach 2016^21^																•	•	**•**	•	•	0
Lees 2011^47^						•	**•**	•	•	•											20
Lesar 2003^48^						•	•	•	•	•											40
Ligi 2010^31^						•	•	•	•	•											100
McFadzean 2023^61^						•	•	•	•	•											40
Meisel 2007^49^						•	•	•	•	•											20
Mondal 2022^62^						•	•	•	•	•											60
Mutter 2003^22^																•	•	•	•	•	0
Patel 2020^50^						•	•	•	•	•											60
Pereira 2020^51^						•	•	•	•	•											80
Ramsay 2014^32^																•	•	•	•	•	100
Rappaport 2011^33^						•	•	•	•	•											100
Robbins 2013^52^											•	•	•	•	•						60
Rungvivatjarus 2020^34^						•	•	•	•	•											100
Russ 2020^53^						•	•	•	•	•											60
Schnipper 2018^65^						•	•	•	•	•											80
Styles 2019^55^, ^59^						•	•	•	•	•											20
Subramanyam 2016^56^						•	•	•	•	•											40
Sullivan 2013^57^						•	•	•	•	•											60
Trap 2018^35^											•	•	•	•	•						100
Triller 2014^58^						•	•	•	•	•											40
Trivedi 2020^66^						•	•	•	•	•											40
Wong 2018^60^						•	•	•	•	•											40

*Note*: Green = Yes, red = No. 1.1. Is the qualitative approach appropriate to answer the research question? 1.2. Are the qualitative data collection methods adequate to address the research question? 1.3. Are the findings adequately derived from the data? 1.4. Is the interpretation of results sufficiently substantiated by data? 1.5. Is there coherence between qualitative data sources, collection, analysis, and interpretation? 3.1. Are the participants representative of the target population? 3.2. Are measurements appropriate regarding both the outcome and intervention (or exposure)? 3.3. Are there complete outcome data? 3.4. Are the confounders accounted for in the design and analysis? 3.5. During the study period, is the intervention administered (or exposure occurred) as intended? 4.1. Is the sampling strategy relevant to address the research question? 4.2. Is the sample representative of the target population? 4.3. Are the measurements appropriate? 4.4. Is the risk of nonresponse bias low? 4.5. Is the statistical analysis appropriate to answer the research question? 5.1. Is there an adequate rationale for using a mixed‐methods design to address the research question? 5.2. Are the different components of the study effectively integrated to answer the research question? 5.3. Are the outputs of the integration of qualitative and quantitative components adequately interpreted? 5.4. Are divergences and inconsistencies between quantitative and qualitative results adequately addressed? 5.5. Do the different components of the study adhere to the quality criteria of each tradition of the methods involved?
